# Combination of Endothelial-Monocyte-Activating Polypeptide-II with Temozolomide Suppress Malignant Biological Behaviors of Human Glioblastoma Stem Cells via miR-590-3p/MACC1 Inhibiting PI3K/AKT/mTOR Signal Pathway

**DOI:** 10.3389/fnmol.2017.00068

**Published:** 2017-03-13

**Authors:** Wei Zhou, Libo Liu, Yixue Xue, Jian Zheng, Xiaobai Liu, Jun Ma, Zhen Li, Yunhui Liu

**Affiliations:** ^1^Department of Neurosurgery, Shengjing Hospital of China Medical UniversityShenyang, China; ^2^Liaoning Research Center for Translational Medicine in Nervous System DiseaseShenyang, China; ^3^Department of Neurobiology, College of Basic Medicine, China Medical UniversityShenyang, China; ^4^Key Laboratory of Cell Biology, Ministry of Public Health of China, and Key Laboratory of Medical Cell Biology, Ministry of Education of China, China Medical UniversityShenyang, China

**Keywords:** EMAP-II, TMZ, GSCs, autophagy, microRNAs, MiR-590-3p, MACC1

## Abstract

This study aims to investigate the effect of Endothelial-Monocyte-Activating Polypeptide-II (EMAP-II) combined with temozolomide (TMZ) upon glioblastoma stem cells (GSCs) and its possible molecular mechanisms. In this study, combination of EMAP-II with TMZ inhibited cell viability, migration and invasion in GSCs, and autophagy inhibitor 3-methyl adenine (3-MA) and chloroquine (CQ) partly reverse the anti-proliferative effect of the combination treatment. Autophagic vacuoles were formed in GSCs after the combination therapy, accompanied with the up-regulation of LC3-II and Beclin-1 as well as the down-regulation of p62/SQSTM1. Further, miR-590-3p was up-regulated and Metastasis-associated in colon cancer 1 (MACC1) was down-regulated by the combination treatment in GSCs; MiR-590-3p overexpression and MACC1 knockdown up-regulated LC3-II and Beclin-1 as well as down-regulated p62/SQSTM1 in GSCs; MACC1 was identified as a direct target of miR-590-3p, mediating the effects of miR-590-3p in the combination treatment. Furthermore, the combination treatment and MACC1 knockdown decreased p-PI3K, p-Akt, p-mTOR, p-S6 and p-4EBP in GSCs; PI3K/Akt agonist insulin-like growth factor-1(IGF-1) partly blocked the effect of the combination treatment. Moreover, *in vivo* xenograft models, the mice given stable overexpressed miR-590-3p cells and treated with EMAP-II and TMZ had the smallest tumor sizes, besides, miR-590-3p + EMAP-II + TMZ up-regulated the expression level of miR-590-3p, LC3-II and Beclin-1 as well as down-regulated p62/SQSTM1. In conclusion, these results elucidated anovel molecular mechanism of EMAP-II in combination with TMZ suppressed malignant biological behaviors of GSCs via miR-590-3p/MACC1 inhibiting PI3K/AKT/mTOR signaling pathway, and might provide potential therapeutic approaches for human GSCs.

## Introduction

Glioblastoma (GBM) is the most common and malignant primary brain tumor in adults. Despite advances in clinical therapies and technologies, the median survival time of GBM patients is only 12–15 months (Mendez et al., 2001; Tso et al., 2015). Glioblastoma stem cells (GSCs) are a neoplastic subpopulation of glioma cells with the potentials of infinite proliferation, self-renewal and multiple differentiation (Cao et al., 2010; Mineo et al., 2016). GSCs are involved in GBM development, therapeutic resistance and recurrence have been confirmed (Auffinger et al., 2015). Therefore, GSCs are considered to be an important therapeutic target for GBM.

Endothelial-Monocyte-Activating Polypeptide-II (EMAP-II) is a tumor-derived cytokine isolated from methylcholanthrene A (Meth A) transformed fibrosarcoma, has various biological functions (Kao et al., 1994). Low-dose EMAP-II can increase the blood-tumor barrier (BTB) permeability by down-regulating the expression levels of tight junction associated proteins (Li et al., 2015). EMAP-II demonstrates significant antitumor activity against pancreatic ductal adenocarcinoma cells and exhibits antitumor effects in prostate adenocarcinoma xenografts (Reznikov et al., 2007; Schwarz et al., 2010). Autophagy is an evolutionarily conserved intracellular lysosomal degradative process in eukaryotic cells for degradation of long-lived proteins and damaged organelles. These cellular proteins and organelles are engulfed in the double-membrane vesicle known as the autophagosome and are transported to the lysosome for degradation (Jiang et al., 2010). Autophagy induction by EMAP-II contributes to its antitumor capacity in human GBM (Liu et al., 2014). Thus, EMAP-II induces GSCs autophagy might play an important role in GBM treatment.

Temozolomide (TMZ) is the second generation oral alkylating agent and becomes the first-line chemotherapeutic agent used for GBM patients (Chen et al., 2014). But as a result of widespread drug resistance for tumor cells, the clinical efficient is less than 45% for TMZ treating GBM patients (Lashford et al., 2002). Accumulating evidences showed that TMZ treatment could induce autophagy (Zhang et al., 2015). One side, TMZ-induced autophagy plays a cytoprotective role of resisting therapy (Zou et al., 2014). On the other side, the cytotoxicity of TMZ to glioma cells was enhanced by autophagy. When combined with thalidomide, a drug could induce autophagy, the cytotoxicity of TMZ to glioma cells was enhanced by autophagy (Gao et al., 2009). Combination of EMAP-II with Rapamycin induces GSCs autophagy and then inhibits the malignant biological behaviors of GSCs (Ma et al., 2015). Therefore, we speculated EMAP-II-induced autophagy might could enhance the antitumor capacity of TMZ.

MicroRNAs (miRNAs, 20–24 nt) are a class of noncoding small molecule RNAs. MiRNAs abnormally express in a variety of tumors and may have the effects of proto-oncogene or anti-oncogene (Stahlhut Espinosa and Slack, 2006). Accumulating researches showed that regulating the expression of miRNAs could enhance the benefits of chemotherapeutics in the treatment of tumors (Tezcan et al., 2014). Overexpressed miR-31 enhances the antitumor activity of TMZ in human GBM cells (Zhou et al., 2015). Moreover, low-dose EMAP-II induces autophagy by down-regulating miR-20a in glioma cells (Chen et al., 2016). Furthermore, miR-590-3p functions as a suppressor of GBM and inhibits cell migration, invasion and epithelial-mesenchymal transition in human GBM cells (Pang et al., 2015). However, whether miR-590-3p is involved in the antitumor activity of combining treatment with EMAP-II and TMZ and its specific mechanism remain unclear.

Metastasis-associated in colon cancer 1 (MACC1) was overexpressed in many tumors, including colon cancer (Arlt and Stein, 2009), human lung cancer (Shimokawa et al., 2011), hepatocellular carcinoma (Sun et al., 2015) and human malignant glioma (Yang et al., 2014). MACC1 gene could regulate various intracellular signal pathways to promote tumorigenesis, malignant development and metastasis (Yao et al., 2015a). In addition, silencing of MACC1 enhance the chemosensitivity of cisplatin in ovarian carcinoma cells (Zhang et al., 2016). However, little has been studied about the role and molecular mechanisms of MACC1 are involved in the antitumor activity of EMAP-II in combination with TMZ.

In the present study, we aimed to determine whether combination of EMAP-II with TMZ could inhibit malignant biological behaviors of GSCs as well as the role of autophagy in the combined therapy. Further, we investigated whether miR-590-3p and MACC1 are involved in the process of EMAP-II combined with TMZ, and explored potential signaling mechanisms.

## Materials and Methods

### Human Tissue Samples and Patient Information

Normal brain tissues (NBTs) and glioma tissues were obtained from patients undergoing surgery at the Department of Neurosurgery, Shengjing Hospital of China. The NBTs tissues were collected from the craniocerebral trauma patients (three cases). Glioma samples were divided into two groups: low grade (grade I–II) and high grade (grade III–IV) according to the WHO classification (six cases). The clinically-relevant details about these patients were shown in Supplementary Table S1. The study procedure was approved by Research Ethics Board at the Shengjing Hospital of China Medical University and the document of the ethical approval for using human tissues was shown in Supplementary Table S1.

### Drugs and Reagents

Dulbecco’s modified Eagle’s medium (DMEM), fetal bovine serum (FBS) and DMEM/F12/Glutamax were purchased from Gibco (Carlsbad, CA, USA). Basic fibroblast growth factor (bFGF), epidermal growth factor (EGF) and 2% B27 were obtained from Life Technologies Corporation (Carlsbad, CA, USA). TMZ, dimethyl sulfoxide (DMSO), 3-methyl adenine (3-MA), chloroquine (CQ) and Z-VAD-fmk (Z-VAD) were purchased from Sigma–Aldrich (St. Louis, MO, USA). EMAP-II and insulin-like growth factor-1 (IGF-1) were purchased from PeproTech (St.Louis, MO, USA). Cell counting kit-8 (CCK-8), DAPI and Lyso-tracker was purchased from Beyotime (Jiangsu, China). Primary antibodies against LC3B (rabbit, polyclonal, ab51520) and MACC1 (rabbit, polyclonal, ab106579) were purchased from Abcam (Cambridge, MA, USA). Primary antibodies against p62/SQSTM1 (rabbit, polyclonal, 18420-1-AP), Beclin-1 (rabbit, polyclonal, 11306-1-AP), PI3K (rabbit, polyclonal, 20584-1-AP), Akt (rabbit, polyclonal, 10176-2-AP) and mTOR (rabbit, polyclonal, 20657-1-AP) were purchased from proteintech (Chicago, USA). Antibodies such as P-PI3K (Tyr458) (rabbit, polyclonal, #4228), p-AKT (Ser473) (rabbit, polyclonal, #9271), p-mTOR (Ser2448) (rabbit, polyclonal, #2971), S6 (rabbit, monoclonal, #2217) and p-S6 (Ser235/236) (rabbit, monoclonal, #4858) were purchased from Cell Signaling Technology (Beverly, MA, USA). Primary antibodies against 4EBP (rabbit, polyclonal, A1248) and p-4EBP (Thr37/46) (rabbit, polyclonal, AP0030) were purchased from ABclonal (Boston, MA, USA). Anti-GAPDH (mouse, monoclonal, sc-365062) and the secondary antibodies conjugated with horseradish peroxidase were bought from Santa Cruz Biotechnology (Santa Cruz, CA, USA).

### Cell Culture and Treatment Conditions

Human GBM cell lines (U87 and U251) and human embryonic kidney (HEK) 293T cells were purchased from Shanghai Institutes for Biological. They were cultured in Dulbecco’s Modified Eagle Medium (DMEM) of high glucose with 10% FBS and were incubated in a 5% CO_2_ humidified incubator at 37°C.

For the experiments, cells were treated with TMZ at different concentrations (50 μM, 100 μM, 200 μM, 400 μM, 600 μM, 800 μM, 1000 μM and 1200 μM, diluted with DMSO) for 24 h, 48 h, and 72 h. As previously reported (Liu et al., 2014), 0.05 nM at 0.5 h were considered to be the optimum concentration and time point of EMAP-II on GSCs, respectively. Furthermore, cells were pretreated with 2 mM 3-MA, 10 μM CQ, 50 μM Z-VAD or 10 nM IGF-1 in different experiments in this study. To test the effect of combination treatment with EMAP-II and TMZ on GSCs, the experiments were divided into four groups: control group, cells were treated with 0.9% sodium chloride (NS) and DMSO; EMAP-II group, cells were treated with 0.05 nM EMAP-II for 0.5 h; TMZ group, cells were treated with 400 μM for 48 h; EMAP-II + TMZ group, cells were pretreated with 0.05 nM EMAP-II for 0.5 h and then plus 400 μM TMZ for 48 h.

### Isolation and Identification of GSCs

GSCs were obtained and isolated as described previously (Yao et al., 2015b). Briefly, GSCs were cultured in DMEM/F-12 medium supplemented with basic fibroblast growth factor (bFGF, 20 ng/ml), epidermal growth factor (EGF, 20 ng/ml) and 2% B27.

### Cell Viability Assay

To evaluate the cytotoxicity of TMZ on glioma cells, CCK-8 assay was performed to determine cell viability. Cells in the logarithmic growth phase were seeded in a 96-well suspension culture plate at 6 × 10^3^ cells/well and incubated for 24 h prior to treatment, then different concentrations of TMZ were added and compared with the DMSO-treated control. At the end of the time point, 10 μl of CCK8 was added to each well and incubated for additional 2 h. Mitochondrial activity is constant for most viable cells and thereby an increase or decrease in the number of viable cells is linearly related to mitochondrial activity, which is the principle of the cell viability assay. CCK8 is based on the WST-8. WST-8 is reduced by dehydrogenases in the cells giving an orange colored formazan. The formazan could reflect the mitochondrial activity of the cells. So, any increase or decrease in viable cell number can be detected by measuring formazan concentration. Optical density (OD) value was finally measured at the wavelength of 450 nm on a microplate reader, and the value was corrected by subtracting the absorbance of control wells that did not contain cells. For the group of pretreatment with EMAP-II and then plus TMZ, the procedure was similar, but cells were treated with 0.05 nM EMAP-II for 0.5 h before TMZ treatment for 48 h or 72 h. The cell viability of transfection cells was also assayed by CCK-8, as previously reported (Zhou et al., 2012).

### Cell Migration and Invasion Assays

The migration and invasion abilities of GSCs were detected using 24-well transwell chambers with 8 μm pore size (Corning Costar). The cells were resuspended in 200 μL serum-free medium and seeded into the upper chamber (without or pre-coated with 500 ng/ml Matrigel solution (BD, Franklin Lakes, NJ, USA) in migration or invasion assay separately) 600 μL of 10% FBS medium was placed in the lower chamber. After incubated for 24 h at 37°C, cells on the top of membrane surface were removed with cotton swabs. Cells on the bottom of the membrane surface were fixed with methanol and glacial acetic acid (mixed at 3:1) for 30 min at room temperature and stained using 10% Giemsa stain for 30 min. Five randomly fields were counted under a microscope and photos were taken.

### Transmission Electron Microscopy

Cells were fixed in ice-cold 2.5% glutaraldehyde overnight at 4°C. After fixation, the samples were post-fixed in 1% osmium tetroxide containing 0.1% potassium ferricyanide for 1 h, and then subjected to the electron microscopy analysis.

### Immunofluorescence Staining

GSCs were stained with LysoTracker Red at a final concentration of 50 nM and incubated in a 5% CO_2_ humidified incubator at 37°C for 1 h. GSCs were harvested by centrifugation and fixed in 4% paraformaldehyde for 30 min. GSCs were blocked with 5% bovine serum albumin for 2 h at room temperature. Following incubation with primary antibody against LC3B at 4°C overnight. The primary antibody was detected with cy3-conjugated anti-rabbit. After that, GSCs were washed with PBS containing 0.1% Tween20 and incubated with 0.5 μg/ml DAPI. The immunofluorescence staining of p62/SQSTM1 was the same with the LC3B only without using LysoTracker Red. The cells were visualized using immunofluorescence microscopy.

### Western Blotting

GSCs were lysed in RIPA buffer supplemented with phenyl-methylsulfonyl chloride (PMSF, 10 ng/ml) on ice and total proteins were extracted from GSCs. Protein concentration was determined using the BCA protein assay kit and equal amounts of proteins were separated in 8%–12% SDS-PAGE and electrophoretically transferred to PVDF membranes. Nonspecific binding was blocked using 5% non-fat milk dissolved in Tris-buffered-saline-Tween (TBST) for 2 h. Subsequently, the membranes were incubated with primary antibodies at 4°C overnight and HRP-conjugated secondary antibodies at room temperature for 2 h. Immunoblots were visualized by ECL detection reagents.

### RNA Extraction and Real-Time PCR

Total RNA were extracted from cells using Trizol reagent (Life Technologies Corporation, Carlsbad, CA, USA). RNA concentration and quality were determined using a Nanodrop Spectrophotometer (ND-100) in the 260/280 nm ratio. We used Taq-Man MicroRNA Reverse Transcription kit and High Capacity cDNA Reverse Transcription Kit for miRNA and mRNA reverse transcription, respectively (Applied Biosystems, Foster City, CA, USA). Quantitative real-time PCR (qRT-PCR) was conducted using TaqMan Universal Master Mix II with TaqMan microRNA assays of miR-590-3p and U6 or TaqMan gene expression assays of MACC1 and GAPDH (Applied Biosystems, Foster City, CA, USA). U6 and GAPDH were used as endogenous control for miRNA and gene expressions, respectively. Expression were normalized to endogenous controls and fold changes were calculated by relative quantification (2^−ΔΔCt^).

### Cell Transfections

MiR-590-3p agomir, miR-590-3p antagomir and their respective non-targeting sequence (negative control, NC) were synthesized by GenePharma in Shanghai, China. GSCs were transfected with miR-590-3p agomir (pre-miR-590-3p), miR-590-3p antagomir (anti-miR-590-3p) or their respective NC using Lipofectamine 2000 reagent (Life Technologies Corporation, Carlsbad, CA, USA). The high transfection efficacy of these could sustain for at least a week from 48 h post-transfection. The time after transfected 48 h was considered as the optimum time in the subsequent experiments. In order to determine the effect of miR-590-3p on GSCs, cells were divided into five groups, Control group, pre-NC group (transfected with negative control), pre-miR-590-3p group (transfected with miR-590-3p agomir), anti-NC group (transfected with negative control) and anti-miR-590-3p (transfected with miR-590-3p antagomir).

In addition, MACC1 was silenced with sh-RNA cloned into pGPU6/GFP/Neo vector (GenePharma). GSCs were transfected with silenced MACC1 plasmids and empty vector transfected using Lipofectamine 3000 reagents (Invitrogen, CA, USA) according to the manufacturer’s instructions. Then GSCs with stable silenced MACC1 were established by using geneticin (G418; Sigma-Aldrich, St. Louis, MO, USA) screening for 4 weeks. To study the effect of MACC1 on GSCs, cells were divided into three groups, Control group, sh-NC group (transfected with sh-NC plasmid), sh-MACC1 group (transfected with sh-MACC1 plasmid).

### Reporter Vectors Constructs and Luciferase Reporter Assays

MACC1 3′-UTR sequences and its mutant of the predicted miR-590-3p binding sites were subcloned into a pMIR-GLOTM Luciferase vector to form MACC1 3′UTR-Wt1 (Wt2) and MACC1 3′UTR-Mut1 (Mut2) (GenePharma, Shanghai, China), respectively. HEK 293T cells were seeded in 96-well plates and co-transfected with MACC1-3′UTR-Wt1 (Wt2) (or MACC1-3′UTR-Mut1 (Mut2)) and pre-NC (or pre-miR-590-3p). The luciferase activities were measured at 48 h after transfection through Dual-Luciferase reporter assay system (Promega, Madison, WI, USA). To explore the implicit mechanism of miR-590-3p in the combination treatment with EMAP-II and TMZ inhibited the malignant biological behavior of GSCs by attenuating MACC1, cells were divided into five groups: control group, anti-NC + sh-NC group (sh-NC stable expressing cells co-transfected with anti-NC), anti-miR-590-3p+sh-NC (sh-NC stable expressing cells co-transfected with anti-miR-590-3p), anti-NC + sh-MACC1 group (sh-MACC1 stable expressing cells co-transfected with anti-NC)and anti-miR-590-3p+sh-MACC1 group (sh-MACC1 stable expressing cells co-transfected with anti-miR-590-3p).

### *In Vivo* Xenograft Study

For the *in vivo* study, GSCs were stably transfected with pre-miR-590-3p. Lentivirus encoding pre-miR-590-3p was generated using pLenti6.3/V5eDEST Gateway Vector Kit (Life Technologies Corporation, Carlsbad, CA, USA). Four-week-old male nude mice were purchased from the National Laboratory Animal Center (Beijing, China). All experiments of the human glioma tissues and nude mice were carried out under the approval of the Administrative Panel on Laboratory Animal Care of Shengjing Hospital. For the *in vivo* study,the incision was closed with stitches and mice were sacrificed by CO_2_ inhalation and death was confirmed by cervical dislocation if they exhibited excessive weight loss of 20% body weight, tumor metastasis, lethargy, or other signs of distress consisted with IACUC standards. There are not vulnerable populations in our study. After 1 week acclimatization, mice were implanted subcutaneously with GSCs or GSCs stably transfected with pre-miR-590-3p into the right flank regions of mice at 2 × 10^6^ cells density. And the tumor-bearing mice were assigned to control group (GSCs treated with 0.9% sodium chloride), EMAP-II + TMZ group (GSCs pretreated with 80 ng/kg EMAP-II i.p. 0.5 h before 50 mg/kg TMZ administration), pre-miR-590-3p (GSCs stably transfected with pre-miR-590-3p), EMAP-II + TMZ + pre-miR-590-3p (pretreated with 80 ng/kg EMAP-II i.p. 0.5 h before 50 mg/kg TMZ administration in pre-miR-590-3p GSCs). Tumor volume was measured with a caliper and calculated as 1/2 × length × width^2^ in mm^3^ every 5 days. Forty five days after implantation, mice were sacrificed and tumors were isolated.

### Statistical Analysis

Data are presented as the mean ± standard deviation (SD). SPSS 18.0 software was used for statistical analysis with the Student’s *t*-test or one-way ANOVA. The *P*-value less than 0.05 was considered statistically significant.

## Results

### EMAP-II in Combination with TMZ Inhibited Cell Viability, Migration and Invasion in GSCs

**Figure 1:**
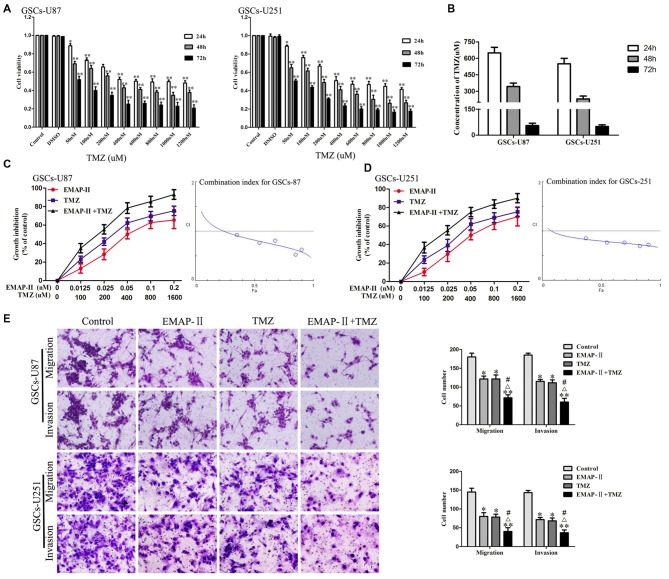
**Cytotoxic effect of temozolomide (TMZ) in glioblastoma stem cells (GSCs)-U87 and GSCs-U251. (A)** GSCs were incubated with various concentration of TMZ (50–1200 μM) and cultured for 24 h, 48 h or 72 h. Cell counting kit-8 (CCK-8) assay were performed to detect the cell viability. Optical density (OD) value of cells was measured by a microplate reader at the wavelength of 450 nm, which was the indicator of cell viability. **(B)** The IC50 values of TMZ in GSCs-U87 and GSCs-U251. Effects of combination treatment with Endothelial-Monocyte-Activating Polypeptide-II (EMAP-II) and TMZ on the cell viability, migration and invasion of GSCs-U87 and GSCs-U251. **(C,D)** Cell viability was detected by CCK-8 assay. GSCs were treated with EMAP-II (0–0.2 nM) for 0.5 h and TMZ (0–1600 μM) for 48 h alone or in combination. The Fa-CI plot shows the combination index value (CI) for each fractional effect. The curves were generated using CompuSyn software. **(E)** Quantification of cell migration and invasion of GSCs after treated with EMAP-II (0.05 nM, 0.5 h), TMZ (400 μM, 48 h), or EMAP-II (0.05 nM, 0.5 h) + TMZ (400 μM, 48 h). Data are presented as the mean ± standard deviation (SD) (*n* = 5, each group). *P < 0.05 vs. Control group, **P < 0.01 vs. Control group, ^#^*P* < 0.05 vs. EMAP-II group, ^Δ^*P* < 0.05 vs. TMZ group.

The cytotoxic effects of TMZ in GSCs were evaluated by using CCK-8 assay. As shown in Figure 1A, the cell viability of GSCs was inhibited by TMZ in a dose and time-dependent manner. The IC50 values of TMZ in GSCs-U87 and GSCs-U251 at 24 h, 48 h and 72 h were shown in Figure 1B. As shown in Figures 1C,D, combination of EMAP-II with TMZ resulted in a significant shift in the cell viability inhibition curve compared with either drug alone, EMAP-II acted synergistically (CI < 1.0) with TMZ to inhibit GSCs-U87 and GSCs-U251 cell viability at almost all combination doses tested. As shown in Figure 1E, the migration and invasion of GSCs in EMAP-II group, TMZ group or EMAP-II + TMZ group were inhibited compared with control group. EMAP-II in combination with TMZ demonstrated even greater inhibitory effect on the migration and invasion of GSCs than single drug, respectively. These results suggested that combination of EMAP-II with TMZ inhibited the malignant biological behaviors of GSCs.

### EMAP-II in Combination with TMZ Enhanced Autophagy in GSCs

**Figure 2:**
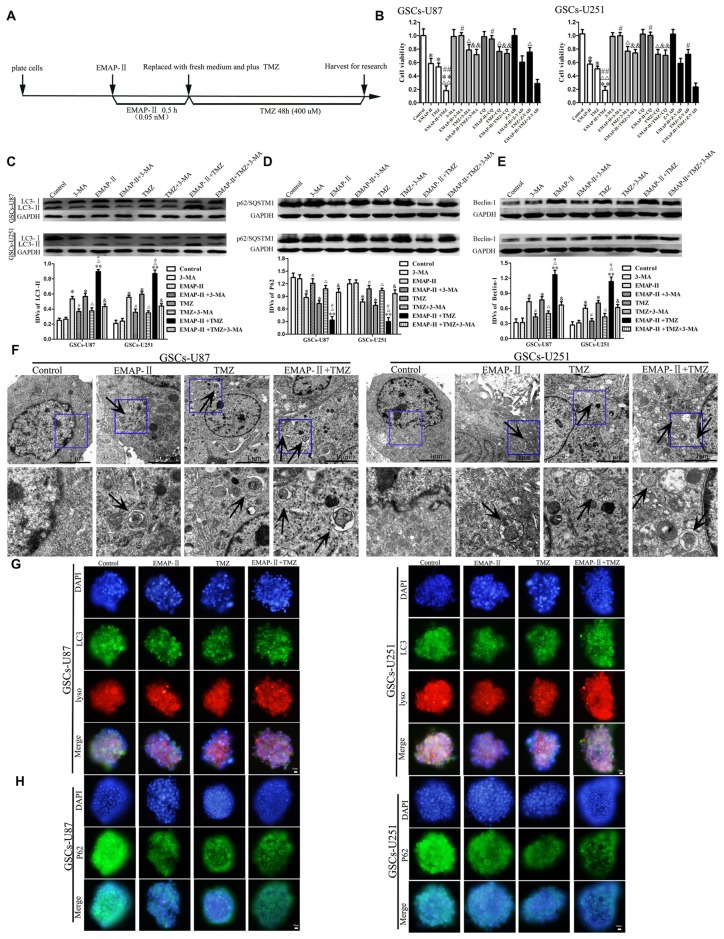
**Combination of EMAP-II with TMZ induced GSCs autophagy. (A)** Timeline of the next combination treatment researches. **(B)** CCK-8 assay were performed to detect the cell viability of GSCs which were incubated with EMAP-II, TMZ or EMAP-II + TMZ and combined with 3-MA, chloroquine (CQ) or Z-VAD, respectively. OD value of cells was measured by a microplate reader at the wavelength of 450 nm. **(C–E)** Western blot analysis was performed to detect the expression of autophagy-related genes. **(F)** Electron microscopy showed ultrastructural features in GSCs treated with EMAP-II, TMZ or EMAP-II + TMZ. Arrows show autophagic vacuoles. **(G)** The colocalization of LC3 and LysoTracker Red in GSCs treated with EMAP-II, TMZ or EMAP-II + TMZ were observed by immunofluorescence assay. Pictures are respective magnification (*n* = 5, each). **(H)** The down-regulation of p62/SQSTM1 in GSCs were observed by immunofluorescence assay after treated with EMAP-II or TMZ or EMAP-II + TMZ. Data are presented as the mean ± SD (*n* = 5, each group) *P < 0.05 vs. Control group, **P < 0.01 vs. Control group, ^#^*P* < 0.05 vs. EMAP-II group, ^##^*P* < 0.01 vs. EMAP-II group, ^Δ^*P* < 0.05 vs. TMZ group, ^ΔΔ^*P* < 0.05 vs. TMZ group, ^&^*P* < 0.05 vs. EMAP-II + TMZ group, ^&&^*P* < 0.01 vs. EMAP-II + TMZ group.

The time line of EMAP-II in combination with TMZ for all the next experiments was shown in Figure 2A. We further investigated whether the inhibitory effect of EMAP-II in combination with TMZ on cell viability was associated with the induced autophagy and apoptosis. GSCs were pretreated with autophagy inhibitor 3-MA, autophagy inhibitor CQ or caspase inhibitor Z-VAD-fmk (Z-VAD). As shown in Figure 2B, 3-MA and CQ pretreatment significantly blocked the inhibitory effect of EMAP-II on the cell viability, and recovered the cell viability to the level in control group. The cell viability of EMAP-II + Z-VAD group was significantly decreased compared with Z-VAD group, while there was no difference between EMAP-II + Z-VAD group and EMAP-II group. 3-MA, CQ and Z-VAD pretreatment partly reverse the anti-proliferative effect of TMZ. The cell viability was inhibited in EMAP-II + TMZ and EMAP-II + TMZ + Z-VAD groups compared with control group, and there was no significant difference between these groups. In addition, the cell viability was increased in EMAP-II + TMZ + 3-MA group or EMAP-II + TMZ + CQ group compared with EMAP-II + TMZ group, suggesting that 3-MA and CQ blocked the inhibitory effect of EMAP-II + TMZ on the cell viability. The above results suggested that inhibitory effects of EMAP-II + TMZ on the cell viability might be associated with cell autophagy in GSCs. In addition, the effects of 3-MA and CQ on the cell viability were consistent and Z-VAD could not reverse the anti-proliferative effect of EMAP-II and EMAP-II + TMZ, so we applied the 3-MA to support the findings in Figure 2B on the western blots assays. As shown in Figures 2C–E, compared with the control group, EMAP-II, TMZ or EMAP-II + TMZ significantly up-regulated LC3-II and Beclin-1 protein expression and down-regulated p62/SQSTM1 protein expression. Combination of EMAP-II with TMZ more significantly increased LC3-II and Beclin-1 protein expression and decreased p62/SQSTM1 protein expression than either EMAP- II or TMZ alone. In addition, the protein expression of LC3-II and Beclin-1 were decreased and the protein expression of p62/SQSTM1 was increased when combined 3-MA with EMAP-II or TMZ. Certainly, 3-MA could also decrease the protein expression of LC3-II and Beclin-1 as well as increased the protein expression of p62/SQSTM1 in the EMAP-II + TMZ group.

As shown in Figure 2F, electron microscopy displayed autophagic vacuoles (AVs) in EMAP-II, TMZ or EMAP-II + TMZ treated GSCs, whereas control cells showed few such features. The AVs increased more obviously in the combination of EMAP-II with TMZ group than either EMAP-II or TMZ alone. As shown in Figure 2G, GSCs were stained with anti-LC3 and LysoTracker Red by immunofluorescence, compared with the control group, high magnification of punctate aggregates were found in GSCs treated with EMAP-II, TMZ or EMAP-II + TMZ. Combination of EMAP-II with TMZ more obviously increased the punctate distribution and density of LC3 in GSCs than either EMAP-II or TMZ alone. In addition, there was a significant overlap between LC3 and lysosomal signals. The immunofluorescence assay of p62/SQSTM1 displayed opposite results as above (Figure 2H). These results suggested that EMAP-II in combination with TMZ enhanced autophagy in GSCs.

### EMAP-II in Combination with TMZ Induced GSCs Autophagy via Up-Regulating miR-590-3p

**Figure 3:**
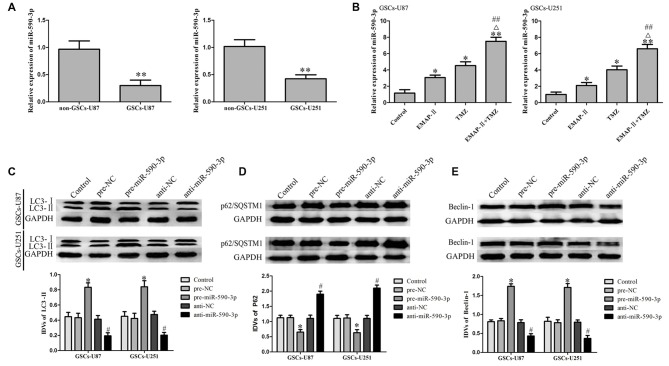
**MiR-590-3p expression in glioblastoma (GBM) cell lines and GSCs. (A)** Expression of miR-590-3p in non-GSCs and GSCs. Data are presented as the mean ± SD (*n* = 5, each group) **P < 0.01 vs. non-GSCs group. **(B)** qRT-PCR analysis for the expression of miR-590-3p in GSCs treated with EMAP-II, TMZ or EMAP-II + TMZ. Data are presented as the mean ± SD (*n* = 5, each group) *P < 0.05 vs. Control group, **P < 0.01 vs. Control group, ^##^*P* < 0.01 vs. EMAP-II group, ^Δ^*P* < 0.05 vs. TMZ group. Overexpression of miR-590-3p induced GSCs autophagy. **(C–E)** Western blot analysis was performed to detect the expression of autophagy-related genes. Data are presented as the mean ± SD (*n* = 5, each group) *P < 0.05 vs. pre-NC group, ^#^*P* < 0.05 vs. anti-NC group.

As shown in Figure 3A, miR-590-3p expression level was significantly lower in GSCs than that in non-GSCs. EMAP-II, TMZ or EMAP-II + TMZ up-regulated the expression level of miR-590-3p compared with the control group. Combination of EMAP-II with TMZ more significantly increased the expression level of miR-590-3p than either EMAP-II or TMZ alone (Figure 3B). As shown in Figures 3C–E, the protein expression level of LC3-II and Beclin-1 significantly up-regulated and the p62/SQSTM1 protein expression level significantly down-regulated in pre-miR-590-3p group compared with pre-NC group, whereas, anti-miR-590-3p group showed the opposite effect. These results revealed that EMAP-II in combination with TMZ induced GSCs autophagy via up-regulating miR-590-3p.

### EMAP-II in Combination with TMZ Induced GSCs Autophagy via Down-Regulating MACC1

**Figure 4:**
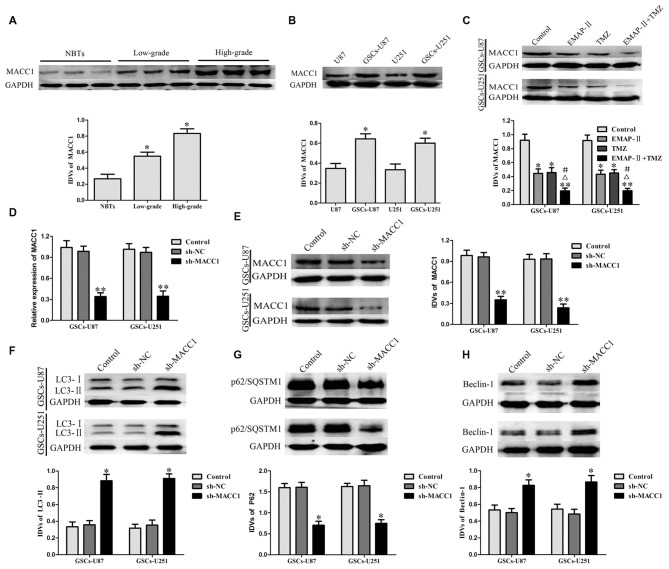
**Metastasis-associated in colon cancer 1 (MACC1) acted as an oncogenic role in glioma tissue and GSCs. (A)** MACC1 protein expression levels in nontumorous brain tissues (NBTs), low-grade glioma tissues (World Health Organization [WHO] I–II), and high-grade glioma tissues (WHO III–IV). Data are presented as the mean ± SD (*n* = 3, each group) *P < 0.05 vs. NBTs group. **(B)** MACC1 protein expression levels in U87, U251 and GSCs. Data are presented as the mean ± SD (*n* = 5, each group) *P < 0.05 vs. non-GSCs group.** (C)** Western blot assay was performed to detect the expression of MACC1 in GSCs treated with EMAP-II, TMZ or EMAP-II + TMZ. Data are presented as the mean ± SD (*n* = 5, each group) *P < 0.05 vs. Control group, **P < 0.01 vs. Control group, ^#^*P* < 0.05 vs. EMAP-II group, ^Δ^*P* < 0.05 vs. TMZ group. MACC1 knockdown induced GSCs autophagy. **(D,E)** The knockdown efficiency of MACC1 by shRNA were detected by qRT-PCR and Western blot assay. **(F–H)** Western blot analysis was performed to detect the expression of autophagy-related genes. Data are presented as the mean ± SD (*n* = 5, each group) *P < 0.05 vs. sh-NC group. Data are presented as the mean ± SD (*n* = 5, each group) *P < 0.05 vs. sh-NC group, **P < 0.05 vs. sh-NC group.

As shown in Figure 4A, compared with NBTs, the protein expression of MACC1 in glioma tissues was significantly increased, in addition, MACC1 expression was positively correlated with the increasing pathological grades of glioma. MACC1 expression levels in GSCs were obviously increased compared with non-GSCs (Figure 4B). These results suggested that MACC1 might play an oncogenic role in GBM development.

EMAP-II, TMZ or EMAP-II + TMZ decreased the protein expression of MACC1 in GSCs compared with the control group. EMAP-II in combination with TMZ more remarkably decreased the protein expression of MACC1 than either EMAP-II or TMZ alone (Figure 4C). The knockdown efficiency of MACC1 by shRNA was shown in Figures 4D,E. As shown in Figures 4F–H, compared with sh-NC group, the protein expression of LC3-II and Beclin-1 in sh-MACC1 group was increased, whereas the p62/SQSTM1 protein expression was decreased. These results showed that EMAP-II in combination with TMZ induced GSCs autophagy via down-regulating MACC1.

### MiR-590-3p Inhibited the Expression of MACC1 by Targeting its 3′-UTR

**Figure 5:**
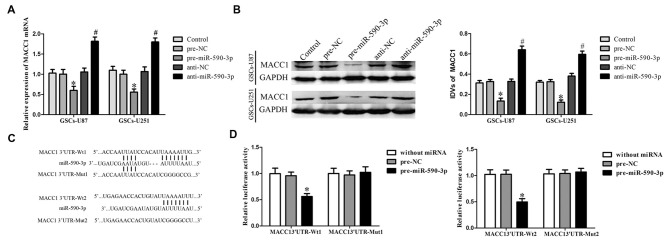
**Overexpression of miR-590-3p inhibited the expression of MACC1 by targeting its 3′-UTR. (A)** Effect of miR-590-3p on the mRNA expression of MACC1 in GSCs. **(B)** Effect of miR-590-3p on the protein expression of MACC1 in GSCs. Data are presented as the mean ± SD (*n* = 5, each group) *P < 0.05 vs. pre-NC group, ^#^*P* < 0.05 vs. anti-NC group. **(C)** The predicted miR-590-3p binding sites in the 3′-UTR region of MACC1 (MACC1-3′-UTR-Wt1 (Wt2)) and the designed mutant sequence (MACC1-3′-UTR-Mut1 (Mut2)) were indicated. **(D)** Luciferase activities were significantly reduced in human embryonic kidney (HEK) 293T cells co-transfected with MACC1-Wt1 (MACC1-Wt2) and pre-miR-590-3p, but not in in HEK 293T cells co-transfected with MACC1-Mut1 (MACC1-Mut2) and pre-miR-590-3p. Data are presented as the mean ± SD (*n* = 5, each group) *P < 0.05 vs. MACC1-Wt1 (MACC1-Wt2) + pre-NC group.

MACC1 was predicted as a potential target gene of miR-590-3p by using the bioinformatics databases (Targetscan, Pictar, miRanda). In order to confirm the predict result, GSCs were transfected with pre-miR-590-3p or anti-miR-590-3p, and assessed mRNA and protein levels of MACC1 by quantitative RT-PCR and Western blot, respectively. MiR-590-3p overexpression decreased the mRNA and protein expression of MACC1, and not surprisingly, inhibition of miR-590-3p increased the mRNA and the protein expression of MACC1 in GSCs (Figures 5A,B). These results suggested that miR-590-3p could inhibit MACC1 expression in GSCs.

Luciferase reporter assay was conducted to illuminate the molecular mechanism. MACC1 was predicted harbor three putative miR-590-3p binding sites in the 3′-UTR by using Targetscan, we choose two of them to perform luciferase assays because their scores are higher. The seeds for miR-590-3p to MACC1 fragment were shown in Figure 5C. As shown in Figure 5D, compared with the pre-NC + MACC1-Wt1(Wt2), luciferase activity was significantly decreased in the pre-miR-590-3p + MACC1-Wt1(Wt2) group, while the luciferase activity in the pre-miR-590-3p + MACC1-Mut1(Mut2) group was not changed comparing with the pre-NC + MACC1-Mut1(Mut2). These results suggested that these two putative binding sites were functional.

### EMAP-II in Combination with TMZ Inhibited the Malignant Biological Behaviors of GSCs via miR-590-3p/MACC1 Inducing Autophagy

**Figure 6:**
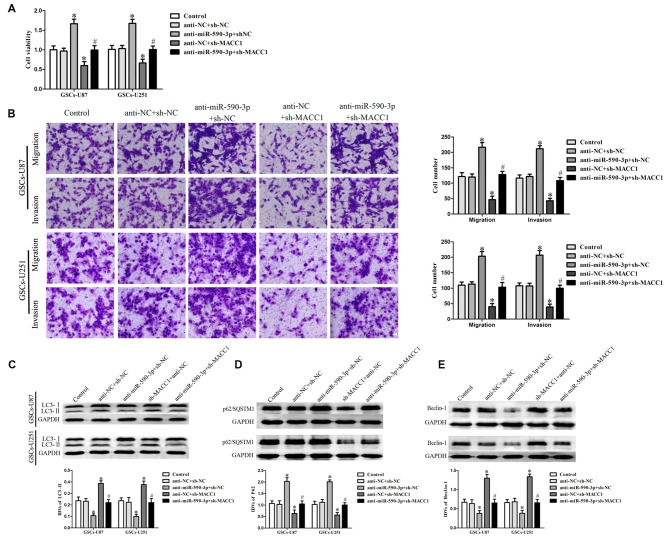
**MACC1 mediated the effect of miR-590-3p in the combination treatment inhibited the malignant biological behaviors of GSCs via inducing autophagy. (A)** Cell viability was detected by CCK8 assay to evaluate the effect of miR-590-3p and MACC1. **(B)** Cell migration and invasion of GSCs was measured by transwell assay to evaluate the effect of miR-590-3p and MACC1. **(C–E)** Western blot assay was performed to detect the expression of autophagy-related genes to evaluate the effect of miR-590-3p and MACC1. Data are presented as the mean ± SD (*n* = 5, each group) *P < 0.05 vs. anti-NC + sh-NC group, ^#^*P* < 0.05 vs. anti-miR-590-3p + anti-NC group.

To confirm whether the effect of miR-590-3p in the combined therapy was mediated by MACC1, MACC1 up-regulation by anti-miR-590-3p was rescued using sh-MACC1 prior to the assessment of the cell viability, migration, invasion and autophagy. As shown in Figure 6A, miR-590-3p down-regulation increased the cell viability of GSCs, whereas MACC1 knockdown reduced the cell viability of these cells. MACC1 knockdown rescued the tumor-promoting effect of miR-590-3p down-regulation on the cell viability of GSCs. Similar to earlier results, miR-590-3p down-regulation promoted the migration and invasion of GSCs, whereas MACC1 knockdown inhibited the migration and invasion of GSCs. MACC1 knockdown rescued the tumor-promoting effect of miR-590-3p down-regulation on the migration and invasion of GSCs (Figure 6B). In addition, miR-590-3p down-regulation decreased the protein expression of LC3-II and Beclin-1 and increased the protein expression of p62/SQSTM1, whereas MACC1 knockdown increased the protein expression of LC3-II and Beclin-1 and decreased the protein expression of p62/SQSTM1. MACC1 knockdown rescued the tumor-promoting effect of miR-590-3p down-regulation on the expression of autophagy related genes of GSCs (Figures 6C–E). The above results revealed that the effect of miR-590-3p in combination of EMAP-II with TMZ induced autophagy inhibited malignant biological behaviors of GSCs was mediated by MACC1.

### EMAP-II in Combination with TMZ Induced GSCs Autophagy Through MACC1 Inhibiting PI3K/AKT/mTOR Signaling Pathway

**Figure 7:**
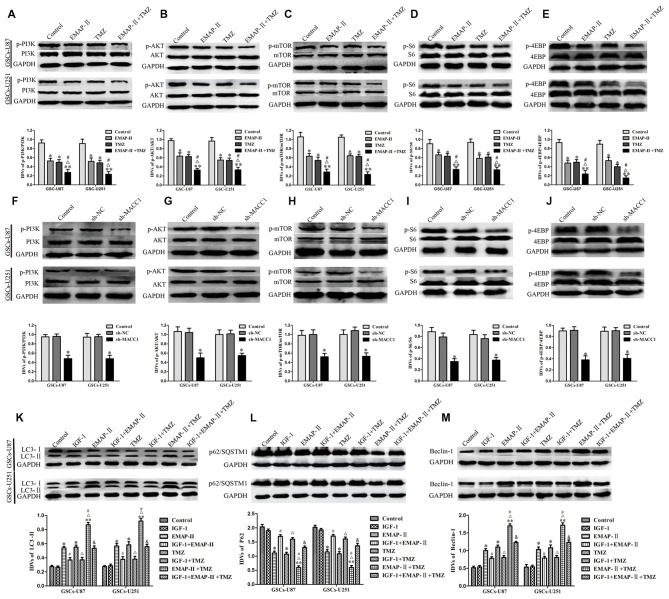
**EMAP-II in combination with TMZ induced GSCs autophagy through MACC1 inhibiting PI3K/AKT/mTOR signaling pathway. (A–E)** GSCs were treated with EMAP-II, TMZ or EMAP-II + TMZ. Western blot assay was performed to detect the PI3K, Akt, mTOR, S6 and 4EBP signal molecules. **(F–J)** MACC1 knockdown inhibited the PI3K/AKT/mTOR pathway. Western blot analysis of the PI3K/AKT/mTOR pathways regulated by MACC1 in GSCs. Data are presented as the mean ± SD (*n* = 5, each group) *P < 0.05 vs. sh-NC group. **(K–M)** PI3K/Akt agonist IGF-1 partly blocked the effect of EMAP-II, TMZ or EMAP-II + TMZ on the expression of autophagy related genes. Data are presented as the mean ± SD (*n* = 5, each group) *P < 0.05 vs. Control group, **P < 0.01 vs. Control group, ^#^*P* < 0.05 vs. EMAP-II group, ^Δ^*P* < 0.05 vs. TMZ group, ^&^*P* < 0.01 vs. EMAP-II + TMZ group.

EMAP-II, TMZ or EMAP-II + TMZ decreased phosphorylated PI3K, Akt, mTOR, S6 and 4EBP in GSCs compared with control group, combination of EMAP-II with TMZ more significantly decreased phosphorylated PI3K, Akt, mTOR, S6 and 4EBP than either EMAP-II or TMZ alone, while total PI3K, Akt, Mtor, S6 and 4EBP were not changed (Figures 7A–E). These results showed that EMAP-II in combination with TMZ more significantly inhibited PI3K/AKT/mTOR signal pathway than either EMAP-II or TMZ alone. As shown in Figures 7F–J, MACC1 knockdown decreased phosphorylated PI3K, Akt, mTOR, S6 and 4EBP in GSCs, while total PI3K, Akt, mTOR, S6 and 4EBP were not change. These results suggested that MACC1 knockdown inhibited the PI3K/AKT/mTOR signal pathway.

To further investigate the role of PI3K/AKT/mTOR signal pathway in the autophagy, PI3K/AKT agonist IGF-1 was used. As shown in Figures 7K–M, the protein expression of LC3-II and Beclin-1 were decreased and the protein expression of p62/SQSTM1 was increased when combined IGF-1 with EMAP-II or TMZ. Certainly, IGF-1 could also decreased the protein expression of LC3-II and Beclin-1 as well as increased the protein expression of p62/SQSTM1 in the EMAP-II + TMZ group. These above results demonstrated that combination of EMAP-II with TMZ induced GSCs autophagy through MACC1 inhibiting PI3K/AKT/mTOR signal pathway.

### Combination Treatment with EMAP-II, TMZ and miR-590-3p Suppressed Tumor Growth *In Vivo*

**Figure 8:**
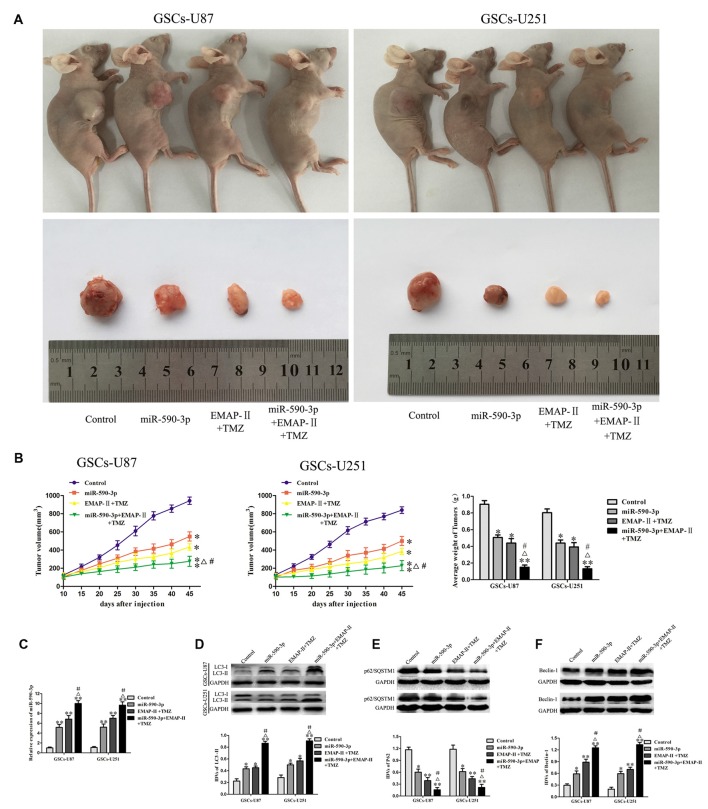
**Combination treatment with EMAP-II, TMZ and miR-590-3p suppressed tumor growth *in vivo*. (A)** Representative images of mice and tumors removed from the xenografted mice were shown. **(B)** Tumor volume was recorded every 5 days, and the tumor was excised and weighed after 45 days. **(C)** qRT-PCR analysis for the expression of miR-590-3p in tumor tissues. **(D–F)** Western blot analysis was performed to detect the expression of autophagy-related genes in tumor tissues. Data are presented as the mean ± SD (*n* = 5, each group) *P < 0.05 vs. Control group, **P < 0.01 vs. Control group, ^#^*P* < 0.05 vs. miR-590-3p group, ^Δ^*P* < 0.05 vs. EMAP-II + TMZ group.

As shown in Figures 8A,B, the results showed that the tumor sizes were smaller in the miR-590-3p group or EMAP-II + TMZ group compared with the control group. The smallest tumor sizes were observed in the miR-590-3p + EMAP-II + TMZ group. Compared with the miR-590-3p group or EMAP-II + TMZ group, the tumor sizes were smaller in the miR-590-3p + EMAP-II + TMZ group. These results showed that miR-590-3p overexpression and combination of EMAP-II with TMZ significantly suppressed tumor growth *in vivo*, in addition, miR-590-3p overexpression enhanced the tumor suppressive effect of combination treatment with EMAP-II and TMZ.

As shown in Figure 8C, the expression level of miR-590-3p in tumor tissues were up-regulated in miR-590-3p group, EMAP-II + TMZ group or miR-590-3p + EMAP-II + TMZ group compared with the control group. Compared with the miR-590-3p group or EMAP-II + TMZ group, the expression level of miR-590-3p in tumor tissues were significantly up-regulated in miR-590-3p + EMAP-II + TMZ group. As shown in Figures 8D–F, compared with the control group, miR-590-3p, EMAP-II + TMZ or miR-590-3p + EMAP-II + TMZ significantly up-regulated LC3-II and Beclin-1 protein expression and down-regulated p62/SQSTM1 protein expression in tumor tissues. Compared with miR-590-3p group or EMAP-II + TMZ group, miR-590-3p + EMAP-II + TMZ significantly up-regulated LC3-II and Beclin-1 protein expression and down-regulated p62/SQSTM1 protein expression in tumor tissues. All the results above demonstrated that miR-590-3p levels and autophagy were associated with the tumor growth.

## Discussion

**Figure 9:**
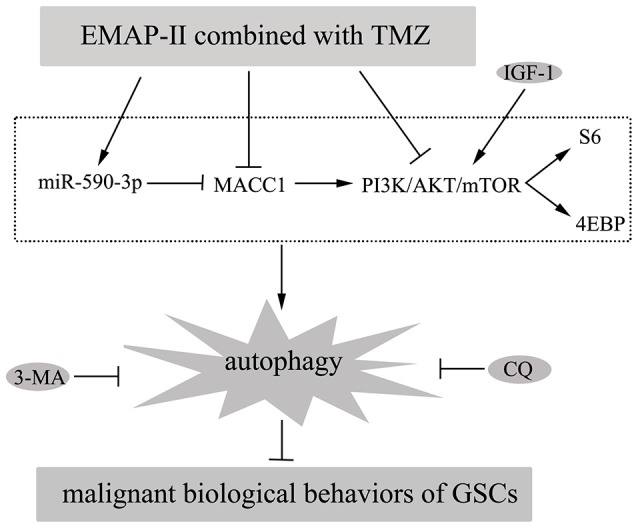
**Diagrammatic presentation of the mechanism of EMAP-II in combination with TMZ suppressed malignant biological behaviors of GSCs via miR-590-3p/MACC1 inhibiting PI3K/AKT/mTOR signaling pathway**.

In this study, we demonstrated that combination of EMAP-II with TMZ inhibited malignant biological behaviors of GSCs by inducing autophagy. Further, miR-590-3p was up-regulated and MACC1 was down-regulated by the combined therapy; MiR-590-3p overexpression and MACC1 knockdown induced GSCs autophagy; MACC1 was confirmed to be the target of miR-590-3p and MACC1 mediated the effects of miR-590-3p in the combined therapy. Furthermore, EMAP-II in combination with TMZ inhibited PI3K/AKT/mTOR signal pathway; MACC1 knockdown also inhibited PI3K/AKT/mTOR signal pathway. The *in vivo* study showed that nude mice carrying overexpressed miR-590-3p cells and treated with EMAP-II and TMZ produced the smallest tumors. The mechanism underlying the suppression of GSCs by EMAP-II in combination with TMZ is schematically presented in Figure 9.

TMZ is the first-line chemotherapeutic drug for GBM patients, but the efficacy of using TMZ alone is limited (Santoni et al., 2012). Accumulating evidences showed that combination of some drugs with TMZ enhanced the antitumor capacity of TMZ in human glioma cells or GSCs, such as metformin (Soritau et al., 2011), demethoxycurcumin (Shi et al., 2015) and olive leaf extract (Tunca et al., 2012). EMAP-II could suppress endothelial cell adhesion to fibronectin, induce endothelial cell apoptosis, exert antiangiogenic effects (Berger et al., 2000; Schwarz et al., 2005). In pancreatic cancer, combination of EMAP-II with bortezomib has anti-proliferative and pro-apoptotic effects (Awasthi et al., 2010). Our previous works showed that 0.05 nM EMAP-II significantly inhibited the cell viability of GSCs at 0.5 h (Liu et al., 2014). In this study, our results showed that the cell viability of GSCs was decreased by TMZ in a dose and time-dependent manner. A previous study demonstrated that 400 μM TMZ inhibited the cell viability of nearly 50% at 48 h for GSCs (Yu et al., 2015b). In addition, combination of EMAP-II with TMZ inhibited the cell viability at almost all combination doses tested. The statistical combination index (CI) was determined for the dual therapy to determine whether combination therapy was synergistic (CI < 1), additive (CI = 1), or antagonistic (CI > 1). We found that EMAP- II acted synergistically (CI < 1.0) with TMZ to inhibit the cell viability of GSCs at almost all combination doses tested. Our results also demonstrated that combination of EMAP-II with TMZ inhibited cell migration and invasion towards GSCs. Thus, combination of EMAP-II and TMZ inhibited malignant biological behaviors of GSCs.

Autophagy can lead to either cancer cell survival or cell death, depending on the cellular context (Carew et al., 2008; Gewirtz, 2014). Some previous reports stated that CQ and its analogs enhance TMZ cytotoxicity in glioma by blocking autophagy (Golden et al., 2014; Rosenfeld et al., 2014). However, according to other studies, various therapeutic drugs could enhance autophagic cell death in glioblastomas, such as thalidomide (Gao et al., 2009) and vitamin (Bak et al., 2016). Several previous studies suggested that EMAP-II inhibited the cell viability of GSCs via inducing autophagy rather than inducing apoptosis (Ma et al., 2015; Chen et al., 2016). In addition, TMZ-induced autophagy and apoptosis inhibited the cell viability of human glioma cells (Chen et al., 2015; Yu et al., 2015b). Our research results are consistent with these studies. We also found that 3-MA and CQ pretreatment significantly blocked the inhibitory effect of EMAP-II + TMZ on the cell viability, while Z-VAD pretreatment could not reverse the anti-proliferative effect of EMAP-II + TMZ. In order to further define the effect of autophagy in combination of EMAP-II and TMZ inhibited malignant biological behaviors of GSCs, several assays were performed. Western blot assays showed that combination of EMAP-II with TMZ more significantly increased the protein expression of LC3-II and Beclin-1 as well as decreased the protein expression of p62/SQSTM1 than either EMAP-II or TMZ alone. The immunofluorescence assay of LC3-II and p62/SQSTM1 displayed similar results with the western blot assays. The electron microscopy displayed that autophagic vacuoles increased more obviously in the combination treatment. Our results suggested that the combination of EMAP-II with TMZ induced GSCs autophagy and thereby inhibited malignant biological behaviors of GSCs.

There was ample evidence that miRNAs are associated with cell proliferation, migration, invasion and autophagy (Ambros, 2004; Gammell, 2007; Kim Y. et al., 2015). MiRNAs are also involved in the antineoplastic process of chemotherapeutic drugs in various types of cancer. MiR-15a/16 induces autophagy by mTORC2 enhances the chemosensitivity of camptothecin in hela cells (Huang et al., 2015b). Overexpression of miR-193b promotes autophagy and non-apoptotic cell death and thereby significantly impedes the ability of esophageal cancer cells to recover following 5-fluorouracil (5-FU) treatment (Nyhan et al., 2016). MiR-590-3p functions as a suppressor of GBM and inhibits cell migration, invasion and epithelial-mesenchymal transition by targeting ZEB1 and ZEB2 in human GBM cells (Pang et al., 2015). Our present data indicated that combination of EMAP-II with TMZ more significantly increased the expression level of miR-590-3p than single drug, respectively. To verify the exact mechanism of miR-590-3p was involved in the combined therapy, the autophagy related genes were detected by western blot assays. We found that miR-590-3p overexpression up-regulated the protein expression levels of LC3-II and Beclin-1 and down-regulated the protein expression level of p62/SQSTM1. We might draw a conclusion that combination of EMAP-II with TMZ induced autophagy via up-regulating miR-590-3p.

MACC1 has been discovered to be related to the cell proliferation, invasion and metastasis in various tumors (Stein et al., 2009; Yao et al., 2015a). MACC1 inhibits the cell apoptosis by targeting the HGF/c-MET/PI3K/AKT signaling pathway in hepatocellular carcinoma (Yao et al., 2015a). MiR-338-3p suppresses epithelial-mesenchymal transition in gastric cancer cells by targeting MACC1/Met/Akt pathway (Huang et al., 2015a). MACC1 promoted the Warburg effect via the PI3K/AKT signaling pathway, which enhanced the resistance to trastuzumab in gastric cancer (Liu et al., 2016). Our results showed that MACC1 played an oncogenic role in GBM and GSCs. Previous studies verified that silencing of MACC1 enhance the apoptosis and growth inhibitory rates of U251 glioma cells, and thereby increase their sensitivity to DDP chemotherapy (Shang et al., 2015). In this study, we found that combination of EMAP-II with TMZ more significantly decreased the protein expression of MACC1 than either EMAP-II or TMZ alone. To verify the exact mechanism of MACC1 was involved in the combined therapy, the autophagy related genes were detected by western blot assays. We found that MACC1 knockdown up-regulated the protein expression levels of LC3-II and Beclin-1 and down-regulated the protein expression level of p62/SQSTM1. MACC1 acted as a target gene for miRNAs, and it has been reported that the expression of MACC1 was down-regulated by miR-143 inhibited the cell migration and invasion in colorectal cancer (Zhang et al., 2012) and MACC1 was down-regulated by miR-200a inhibited the hepatocellular carcinoma cell proliferation and migration (Feng et al., 2015). In our study, miR-590-3p overexpression decreased the expression of MACC1, in addition, MACC1 was identified as a putative target of miR-590-3p. Based on these results, we wondered combination of EMAP-II with TMZ inhibited malignant biological behaviors of GSCs via miR-590-3p inducing autophagy was partially dependent on the regulation of MACC1. Several studies were performed to support this hypothesis. We found that MACC1 down-regulation rescued the tumor-promoting effect of miR-590-3p low-expression on the cell viability, migration, invasion and autophagy of GSCs. Thus, miR-590-3p up-regulation was significant in the effect of EMAP-II in combination with TMZ induced autophagy inhibited malignant biological behaviors of GSCs, which was partly through the inhibition of MACC1 expression.

PI3K/AKT/mTOR signaling pathway plays important roles in regulating cell proliferation, migration, invasion and autophagy (Heras-Sandoval et al., 2014; Yousef et al., 2016). The mammalian target of rapamycin (mTOR) is a key regulator of the initiation of autophagy (Maiese et al., 2013; Roy et al., 2014). mTOR is activated by the PI3K/AKT pathway and regulates ribosomal biogenesis and protein synthesis by phosphorylating the downstream effectors, S6 and 4EBP (Misra and Pizzo, 2012). EMAP-II induces autophagy through PI3K/AKT/mTOR signaling pathway inhibits malignant biological behaviors of human GBM cells and GSCs (Ma et al., 2015). It has been proposed that PI3K/AKT/mTOR pathway could play the dual roles of responding to TMZ treatment for GBM. On one hand, Lenz G and colleagues found that acute treatment with TMZ induces the sustained inhibition of Akt-mTOR, which produced a transient induction of autophagy, leading to cell resistance of the therapy (Filippi-Chiela et al., 2015). On the other hand, Yu et al.’s (2015a) group demonstrated that TMZ inhibits the cell proliferation and promotes apoptosis through inhibiting the PI3K/AKT/mTOR signaling pathway, and the dual PI3K-mTOR inhibitor NVP-BEZ235 enhances the cytotoxicity of TMZ for GBM. In the study, we found that combination of EMAP-II with TMZ more significantly decreased phosphorylated PI3K, Akt, mTOR, S6 and 4EBP than either EMAP-II or TMZ alone. In addition, MACC1 knockdown also decreased phosphorylated PI3K, Akt, mTOR, S6 and 4EBP. PI3K/Akt agonist IGF-1 partly blocked the effect of combination treatment on the expression of autophagy related genes. Our previous results showed that combination of EMAP-II with TMZ more significantly decreased the protein expression of MACC1 and MACC1 knockdown induced GSCs autophagy. Therefore, combination of EMAP-II with TMZ induced GSCs autophagy through MACC1 inhibited PI3K/AKT/mTOR signaling pathway.

A previous report established that tumors derived from GSCs were significantly suppressed in EMAP-II-treated nude mice (Liu et al., 2014), and not surprisingly, TMZ could also suppress tumor growth *in vivo* xenograft models (Kim S.-S. et al., 2015). Our *vivo* tumor xenografts study demonstrated that the combination of EMAP-II with TMZ significantly suppressed tumor growth. Overexpression of miR-590-3p also significantly suppressed tumor growth. In addition, the smallest tumor sizes were observed in the miR-590-3p + EMAP-II + TMZ group. Moreover, in order to clarify the mechanism of the reduction in tumor growth by the combination therapy, qRT-PCR and western blots were used. We found that the expression level of miR-590-3p in tumor tissues were up-regulated in miR-590-3p + EMAP-II + TMZ group compared with the miR-590-3p group or EMAP-II + TMZ group, besides, miR-590-3p + EMAP-II + TMZ significantly up-regulated LC3-II and Beclin-1 protein expression and down-regulated p62/SQSTM1 protein expression in tumor tissues compared with the miR-590-3p group or EMAP-II + TMZ group. These results showed that miR-590-3p levels and autophagy are associated with the tumor size.

In conclusion, our results demonstrated that miR-590-3p was up-regulated by the combination of EMAP-II with TMZ inhibited the expression of MACC1 induced GSCs autophagy through the inhibition of PI3K/AKT/mTOR pathway, and thereby inhibited malignant biological behaviors of GSCs, providing an attractive new therapeutic approach for human GSCs.

## Author Contributions

YL, YX and LL: conceived and designed the experiments. WZ, JZ and XL: performed the experiments. WZ, LL and ZL: analyzed the data. WZ, LL and YX: wrote the manuscript. All authors listed, have made substantial, direct and intellectual contribution to the work and approved it for publication.

## Conflict of Interest Statement

The authors declare that the research was conducted in the absence of any commercial or financial relationships that could be construed as a potential conflict of interest.
