# Supplementary material for: Combination of Endothelial-Monocyte-Activating Polypeptide-II with Temozolomide Suppress Malignant Biological Behaviors of Human Glioblastoma Stem Cells via miR-590-3p/MACC1 Inhibiting PI3K/AKT/mTOR Signal Pathway
Source: Front Mol Neurosci. 2017 Mar 13;10:68. doi: 10.3389/fnmol.2017.00068 (PMC5346543; doi:10.3389/fnmol.2017.00068)
Supplement: Supplementary file 1 [file Table_1.pdf]

*Supplementary Material*

# Combination of Endothelial-Monocyte-Activating Polypeptide-II with Temozolomide Suppress Malignant Biological Behaviors of Human Glioblastoma Stem Cells via miR-590-3p/MACC1 Inhibiting PI3K/AKT/mTOR Signal Pathway

**Wei Zhou[1,2], Libo Liu[3,4], Yixue Xue[3,4], Jian Zheng[1,2], Xiaobai Liu[1,2], Jun Ma[3,4], Zhen Li[1,2], Yunhui Liu[1,2]***

[1]Department of Neurosurgery, Shengjing Hospital of China Medical University, Shenyang China

[2]Liaoning Research Center for Translational Medicine in Nervous System Disease, Shenyang China

[3]Department of Neurobiology, College of Basic Medicine, China Medical University, Shenyang China

[4] Key Laboratory of Cell Biology, Ministry of Public Health of China, and Key Laboratory of Medical Cell Biology, Ministry of Education of China, China Medical University, Shenyang China

***Correspondence:** Yunhui Liu: liuyh_cmuns@163.com

## 1. Supplementary data

Supplementary Table S1. The clinically-relevant details of patient tissues.
The study was approved by the Ethics Committee of Shengjing Hospital of China Medical University. The grade was identified according to the WHO classification by experienced clinical pathologists.

| Patient number | Age | Sex | WHO Grade | Histology type | Lobe |
|---|---|---|---|---|---|
| 1 | 32 | M | | Craniocerebral trauma | Frontal lobe |
| 2 | 22 | M | | Craniocerebral trauma | Frontal lobe |
| 3 | 38 | F | | Craniocerebral trauma | Frontal lobe |
| 4 | 47 | F | I | Pilocytic astrocytoma | Temporal lobe |
| 5 | 59 | F | I | Pilocytic astrocytoma | Temporal lobe |
| 6 | 45 | M | II | Diffuse astrocytoma | Temporal lobe |
| 7 | 61 | M | IV | Glioblastoma | Frontal lobe |
| 8 | 69 | F | IV | Glioblastoma | Frontal lobe |
| 9 | 59 | M | IV | Glioblastoma | Frontal lobe |
